# A Global Phosphorylation Atlas of Proteins Within Pathological Site of Rotator Cuff Tendinopathy

**DOI:** 10.3389/fmolb.2021.787008

**Published:** 2022-02-15

**Authors:** Yezhou Wang, Jiawei Zhang, Yuan Lin, Shi Cheng, Duanyang Wang, Man Rao, Yuheng Jiang, Xiang Huang, Ruijing Chen, Yong Xie, Pengbin Yin, Biao Cheng

**Affiliations:** ^1^ School of Medicine, Shanghai Tenth People’s Hospital, Tongji University, Shanghai, China; ^2^ School of Rehabilitation, Capital Medical University, Beijing, China; ^3^ China Rehabilitation Research Center, Beijing, China; ^4^ Department of Orthopedics, The Second Affiliated Hospital of Harbin Medical University, Harbin, China; ^5^ Department of Orthopedics, Fourth Medical Center of PLA General Hospital, Beijing, China; ^6^ National Clinical Research Center for Orthopedics, Sports Medicine and Rehabilitation, Beijing, China

**Keywords:** rotator cuff tendinopathy, phosphorylation, Wnt, proteomics, TNF signaling pathway

## Abstract

Rotator cuff tendinopathy (RCT) is the most common cause of shoulder pain, therefore posing an important clinical problem. Understanding the mechanism and biochemical changes of RCT would be of crucial importance and pave the path to targeting novel and effective therapeutic strategies in translational perspectives and clinical practices. Phosphorylation, as one of the most important and well-studied post-translational modifications, is tightly associated with protein activity and protein functional regulation. Here in this study, we generated a global protein phosphorylation atlas within the pathological site of human RCT patients. By using Tandem Mass Tag (TMT) labeling combined with mass spectrometry, an average of 7,741 phosphorylation sites (p-sites) and 3,026 proteins were identified. Compared with their normal counterparts, 1,668 p-sites in 706 proteins were identified as upregulated, while 73 p-sites in 57 proteins were downregulated. GO enrichment analyses have shown that majority of proteins with upregulated p-sites functioned in neutrophil-mediated immunity whereas downregulated p-sites are mainly involved in muscle development. Furthermore, pathway analysis identified NF-κB–related TNF signaling pathway and protein kinase C alpha type (PKCα)–related Wnt signaling pathway were associated with RCT pathology. At last, a weighted kinase-site phosphorylation network was built to identify potentially core kinase, from which serine/threonine-protein kinase 39 (STLK3) and mammalian STE20-like protein kinase 1 (MST1) were proposed to be positively correlated with the activation of Wnt pathway.

## Introduction

Tendinopathy is a term used to describe a complex multi-faceted pathology of the tendon characterized by pain, decline in function, and reduced exercise tolerance. Tendinopathy poses an important clinical problem—particularly in musculoskeletal and sports-related medicine—and accounts for up to 30% of general practice musculoskeletal consultations (Millar et al., 2017). The incidence of shoulder complaints is approximately 11.2 cases per 1,000 patients per year (van der Windt et al., 1995). Patients with rotator cuff tendinopathy (RCT) comprise a sizeable portion of this subpopulation, and RCT is the most common cause of shoulder pain. Rotator cuff disorders are a significant source of morbidity among manual laborers and those whose work involves a great deal of repetitive motion.

Conservative approaches were mostly recommended when the patients first present to the hospital accompanied with RCT (Doiron-Cadrin et al., 2020). Due to a lack of understanding in the exact mechanism, no specific agent targeting on any molecule or biological pathway exists. General approaches, i.e., cryotherapy, NSAIDs, and glucocorticoids, are more likely to be provided, but few are supported by high-quality research-based evidence (Rees et al., 2006). Surgery is the last option. According to the existing guideline, orthopedic surgical referral is obtained if nonoperative therapy fails to provide relief within 6–9 months or a diagnosis of rotator cuff tear is made. Nonetheless, a good prognosis still could barely be guaranteed, not to say that patients have to bear sustainable pain and a significant loss of shoulder range of motion. Therefore, understanding the mechanism and biochemical changes of RCT so as to provide more therapeutic options for this condition is of crucial importance.

Prior research has applied proteomic approach in uncovering the protein level changes along with RCT (Sejersen et al., 2015; Yuan et al., 2021). Matrix metalloproteinases (MMPs) were found to be involved in the pathogenesis of RCT, and a remodeling of extracellular matrix (ECM) was reflected by the alteration of MMPs and collagen expression. In addition, oxidative stress was also considered to be relevant to RCT. Of note, phosphorylation, as one of the most important and well-studied post-translational modifications, is tightly associated with protein activity and is a key point of protein function regulation (Ubersax and Ferrell 2007). Reversible phosphorylation of serine, threonine, and tyrosine residues is a key step in the control of signal transduction pathways (Olsen et al., 2006; Humphrey et al., 2015). Therefore, protein phosphorylation has become a central focus of drug discovery as the result of the identification and validation of promising therapeutic targets such as protein kinases, protein phosphatases, and phosphoprotein binding domains. However, no prior research has provided a phosphorylation atlas of proteins within the pathological site of RCT as compared with the normal site.

## Material and Methods

### Ethics Approval

This study adhered to the Declaration of Helsinki and was approved by Ethics Committee of Shanghai Tenth People’s Hospital (2019-K-167). Written informed consents had been obtained when the participants were included.

### Patients and Diagnosis

Subjects were recruited from patients admitted to the Shanghai Tenth People’s Hospital, Tongji University. Diagnosis of RCT was based on the existing guideline, which takes into account the symptom, physical examination, and MRI reports. Patients with congenital musculoskeletal disease, cancer, or other diseases affecting muscle and tendon are excluded. RCT samples were harvested during the surgery while control tendon samples were obtained from unaffected sites of the same patients.

### Protein Extraction

The sample was taken out from −80°C, and a proper amount of tissue sample was weighed and placed into a mortar precooled with liquid nitrogen, and then fully ground to powder by adding liquid nitrogen. After that, four volumes of lysis buffer (8 M urea, 1% Triton X-100, 1% protease inhibitor cocktail, 1% phosphatase inhibitors, 3 μM trichostatin-A, and 50 mM nicotinamide) was added to the tissue powder, followed by sonication at 30% of maximum power in cycles of 10 s on and 10 s off for 3 times on ice using a high-intensity ultrasonic processor (Scientz). The remaining debris was removed by centrifugation at 12,000×*g* at 4°C for 10 min. Finally, the supernatant was collected and the protein concentration was determined with BCA kit according to the manufacturer’s instructions. Equal amounts (400 μg) of each sample from the same group were pooled and used for the following steps.

### Trypsin Digestion

For digestion, the protein solution was reduced with 5 mM dithiothreitol for 30 min at 56°C and alkylated with 11 mM iodoacetamide for 15 min at room temperature in darkness. The protein sample was then diluted by adding 100 mM tetraethyl-ammonium bromide to urea concentration less than 2 M. Finally, trypsin was added at 1:50 trypsin-to-protein mass ratio for the first digestion overnight and 1:100 trypsin-to-protein mass ratio for a second 4-h digestion.

### Tandem Mass Tag Labeling

After trypsin digestion, peptide was desalted by Strata X C18 SPE column (Phenomenex) and vacuum dried. Peptide was reconstituted in 0.5 M TEAB and processed according to the manufacturer’s protocol for TMT kit (Thermo Fisher). Briefly, 1 U of TMT reagent was thawed and reconstituted in acetonitrile. The peptide mixtures were then incubated for 2 h at room temperature and pooled, desalted, and dried by vacuum centrifugation.

### HPLC Fractionation

The tryptic peptides were fractionated into fractions by high pH reverse-phase HPLC using Thermo Betasil C18 column (5 μm particles, 4.6 mm ID, 250 mm length). Briefly, peptides were first separated with a gradient of 8–32% acetonitrile (pH 9.0) over 60 min into 60 fractions. Then, the peptides were combined into 4 fractions and dried by vacuum centrifuging.

### Affinity Enrichment

For the biomaterial-based post-translational modification enrichment (for phosphorylation), peptide mixtures were first incubated with Fe-IMAC microsphere suspension with vibration in loading buffer (50% acetonitrile/6% trifluoroacetic acid). The Fe-IMAC microspheres with enriched phosphopeptides were collected by centrifugation. To remove nonspecifically adsorbed peptides, the Fe-IMAC microspheres were washed with 50% acetonitrile/6% trifluoroacetic acid and 30% acetonitrile/0.1% trifluoroacetic acid sequentially. To elute the enriched phosphopeptides from the Fe-IMAC microspheres, elution buffer containing 10% NH_4_OH was added and the enriched phosphopeptides were eluted with vibration. The supernatant containing phosphopeptides was collected and lyophilized for LC-MS/MS analysis.

### LC-MS/MS Analysis

The tryptic peptides were dissolved in 0.1% formic acid (solvent A) and directly loaded onto a home-made reversed-phase analytical column (15 cm length, 75 μm i.d.). The gradient was composed of an increase from 6 to 23% solvent B (0.1% formic acid in 98% acetonitrile) over 26 min, 23–35% in 8 min, and climbing to 80% in 3 min then holding at 80% for the last 3 min, all at a constant flow rate of 400 nl/min on an EASY-nLC 1000 UPLC system. The peptides were subjected to NSI source followed by tandem mass spectrometry (MS/MS) in Q Exactive HF-X (Thermo) coupled online to the UPLC. The electrospray voltage applied was 2.0 kV. The m/z scan range was 350–1,800 for full scan, and intact peptides were detected in the Orbitrap at a resolution of 70,000. Peptides were then selected for MS/MS using NCE setting as 28 and the fragments were detected in the Orbitrap at a resolution of 17,500. A data-dependent procedure alternated between one MS scan followed by 20 MS/MS scans with 15.0 s dynamic exclusion. Automatic gain control was set at 5E4. Fixed first mass was set as 100 m/z.

### Database Search

The resulting MS/MS data were processed using Maxquant search engine (v.1.6.6.0). Tandem mass spectra were searched against SwissProt database concatenated with reverse decoy database. Trypsin/P was specified as cleavage enzyme allowing up to 4 missing cleavages. First search range was set to 5 ppm for precursor ions, and main search range was set to 5 ppm and 0.02 Da for fragment ions. Carbamidomethyl on Cys was specified as fixed modification and phosphorylation on Ser, Thr, Tyr, and oxidation on Met were specified as variable modifications. False discovery rate was adjusted to <1% and minimum score for modified peptides was set >40.

### Bioinformatics Analyses

Functional analysis of proteins was determined by gene ontology (GO) enrichment. GO terms for biological process (BP), molecular function (MF), and cellular component (CC) charts were obtained using default statistical parameters. Regarding pathway analysis, first Kyoto Encyclopedia of Genes and Genomes (KEGG) pathways were constructed and the gene set enrichment analysis (GSEA) approach, which took the quantification information into account, was further applied. For inferring protein kinase identification, a method by prior research was applied in our dataset (Abe et al., 2020). Briefly, we adopted iGPS1.0 software for predicting kinase-substrate regulations, which based on the theory of short linear motifs (SLMs) around phosphorylation sites (p-sites) provide the primary specificity. The software utilized GPS2.0 algorithm5 for the prediction of site-specific kinase-substrate relations (ssKSRs) and protein–protein interaction (PPI) information was used as the major contextual factor to filtrate potentially false-positive hits. A “medium” threshold was chosen and the parameter “Interaction” was set to “Exp./String”. Then GSEA method was used to predict kinase activities, in which log-transformed phosphorylation levels (or ratio values) as a rank file and kinase-phosphorylation site regulations were formatted into a gmt file in a sample (or a comparable group). Normalized enrichment scores (NES) of enrichment result were regarded as kinase activity scores. For each kinase, the kinase was predicted as positive if the predominant change of substrates was an increase in phosphorylation and *vice versa*. At last, according to the complicated regulatory relationships, for each comparison group, kinases predicted as positive or negative activity and significantly differential expressed phosphorylation sites were used to constructed kinase-substrate regulatory network.

### Western Blotting

Protein samples were extracted as mentioned previously. Following SDS–polyacrylamide gel electrophoresis, western blotting was performed by standard procedure. An equal amount (40 μg) of each sample was loaded. Antibodies against STLK3 (dilution 1:1,000, #ab128894), MST1 (dilution 1:1,000, #ab245190), *p*-PKCα (dilution 1:1,000, #ab76016), and PKCα (dilution 1:1,000, #ab32376) were purchased from Abcam (Cambridge, MA). Antibody against β-actin (dilution 1:10,000, #66009-1-Ig) was purchased from Proteintech (Wuhan, China).

### Statistical Analysis

**FIGURE 1:**
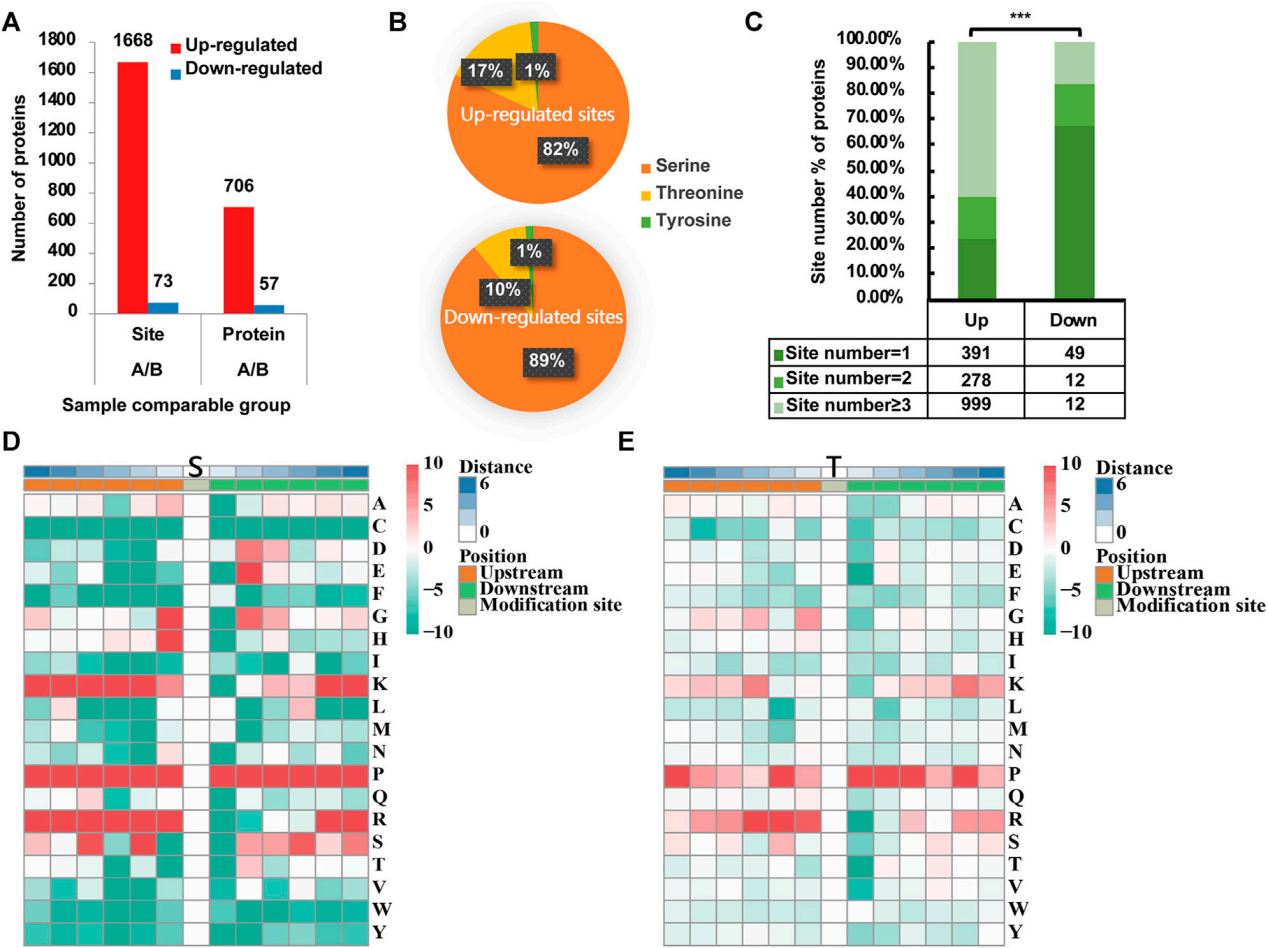
Phosphoproteomic profile in rotator cuff tendinopathy site. **(A)** 1,668 p-sites in 706 proteins were identified as upregulated, while 73 sites in 57 proteins were downregulated. **(B)** Among upregulated sites (upper chart), 1,366 located in serine (S), accounting for 82% of total upregulated sites; 277 (17%) in threonine (T) and 25 (1%) in tyrosine (Y). Comparatively, downregulated sites (lower chart) occurred in 65 S (89%), 7 T (10%), and 1 Y (1%). **(C)** For proteins with upregulated p-sites, 23.44% have 1 site upregulated, 16.67% have 2 sites, and 59.89% have 3 or more sites upregulated. On the contrary, for proteins with downregulated p-sites, the large majority of proteins (67.12%) have only 1 site downregulated. Statistical significance was assessed using Pearson’s χ^2^ test; ***p < 0.001. **(D)** Analysis of the serine p-site motif in the adjacent 6 positions either upstream or downstream (−6∼+6). **(E)** Tyrosine p-site motif in the adjacent 6 positions either upstream or downstream (−6∼+6).

GraphPad Prism 8 was used to perform statistical analysis. The constitutions of proteins with up/downregulated p-sites (Figure 1C) were assessed using Pearson’s χ^2^ test. In western blotting validation, the relative protein expression was normalized to β-actin using ImageJ software and then analyzed by paired samples *t*-test. A *p*-value <0.05 was considered significant at 0.05 level (*p < 0.05, **p < 0.01, ***p < 0.001).

## Results

### Characteristics of the Included Patients

A total of nine patients diagnosed as RCT were recruited from the Tenth People’s Hospital of Tongji University (Table 1), among which 6 were female and 3 were male. The age ranged from 41 to 73 years, with a median of 68. The age range of included patients mirrored the vulnerable population reported by prior studies. Given that the patients’ samples were taken during operation, all included patients have experienced duration of disease over 6 months and the longest one up to 72 months. No patients have complained a clear history of shoulder injury and no one has ever been smoking. Most patients were not accompanied with any comorbidities with two expectations who claimed a history of stroke and hypertension, respectively. These two patients regularly took statins and irbesartan to treat their respective conditions.

**TABLE 1:** Demographic characteristics of included patients.

No	Gender	Age (years)	Height (cm)	Weight (kg)	Diagnosis	A clear history of injury (Y/N)	Disease duration (months)	Smoking (Y/N)	Any comorbidities (Y/N/diagnosis)	Medication history (Y/N/medication)
1	Male	41	176	66	RCT	N	12	N	N	N
2	Female	59	150	50	RCT	N	10	N	N	N
3	Female	72	163	80	RCT	N	6	N	N	N
4	Male	50	173	72.5	RCT	N	72	N	N	N
5	Female	68	156	61.5	RCT	N	60	N	Stroke	Statins
6	Male	77	165	62.5	RCT	N	12	N	N	N
7	Female	73	158	49.5	RCT	N	8	N	Hypertension	Irbesartan
8	Female	70	166	70	RCT	N	18	N	N	N
9	Female	62	158	49	RCT	N	12	N	N	N

### Phosphoproteomic Profile in RCT Site

Based on the quantification of phosphorylation within tendinopathy site versus normal tendon, we set a ratio >1.3 as upregulation and <0.77 as downregulation thresholds. In total, 1,668 phosphorylation sites (p-sites) in 706 proteins were identified as upregulated, while 73 sites in 57 proteins were downregulated (Figure 1A). Among upregulated sites, 1,366 located in serine (S), accounting for 82% of total upregulated sites (Figure 1B); 277 (17%) in threonine (T) and 25 (1%) in tyrosine (Y). Comparatively, downregulated sites occurred in 65 S (89%), 7 T (10%), and 1 Y (1%). Moreover, for those proteins with upregulated p-sites, 23.44% have one site upregulated, 16.67% have 2 sites, and 59.89% have 3 or more cites upregulated (Figure 1C). On the contrary, for proteins with downregulated p-sites, the large majority of proteins (67.12%) have only 1 site downregulated. Furthermore, analysis of the serine p-sites revealed that the phosphorylation motif showed a strong preference for proline (P) in the adjacent 6 positions either upstream or downstream (−6∼+6). Moreover, lysine (K) and arginine (R) are more likely to present in the upstream (−6∼0). As for threonine p-sites, similar motifs were seen although the preference was not as significant as the motifs for serine p-sites (Figure 1D, E).

### GO Enrichment Analysis

**FIGURE 2:**
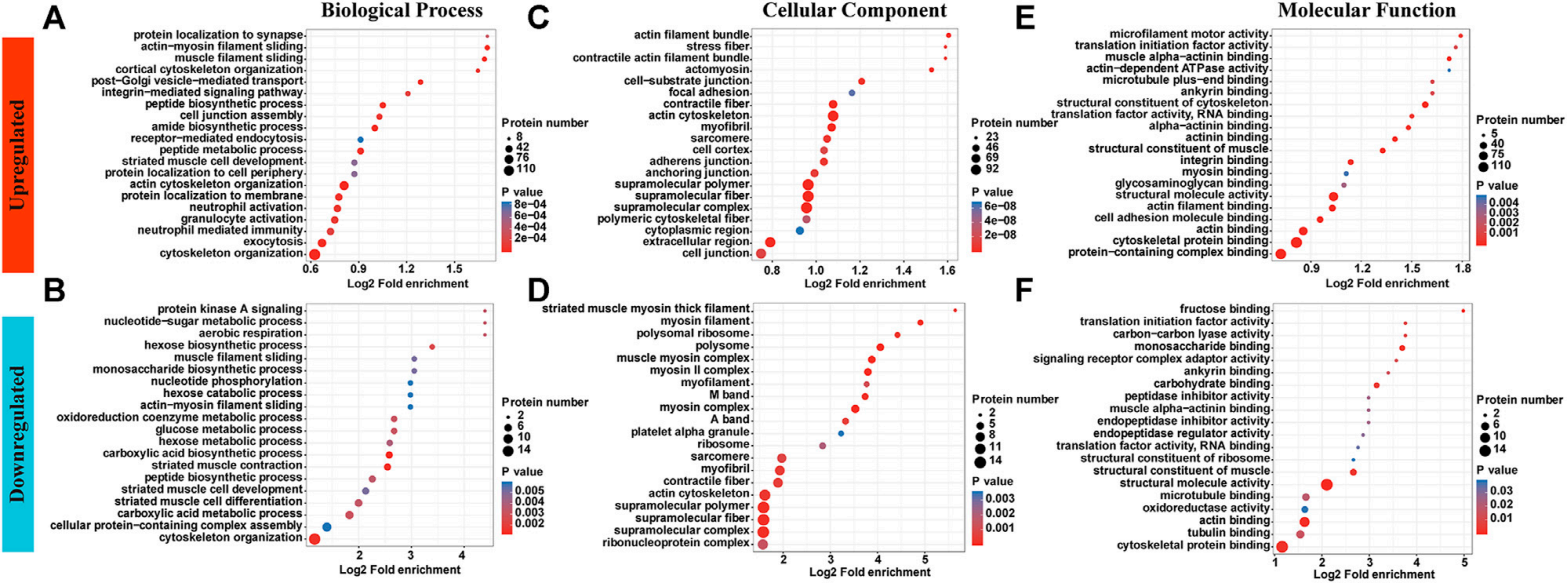
GO enrichment analyses showed different enrichment patterns across two groups. **(A,B)** Upregulated p-site proteins were highly enriched in neutrophil or granulocyte activation, and neutrophil-mediated immunity. While proteins were enriched in muscle filament sliding, muscle cell development and differentiation showed decreased levels of phosphorylation. **(C,D)** Most proteins identified are tendon/muscle assemblies, be those with increased phosphorated p-sites or with decreased p-sites. **(E,F)** With respect to molecular function, a significant enrichment in integrin binding was witnessed for upregulated p-site proteins. In addition, the rest are enriched in basic muscle/tendon functions.

GO enrichment analyses showed different enrichment patterns across two groups. Upregulated p-site proteins were most highly enriched in cytoskeletal organization and exocytosis, followed by inflammation-related neutrophil or granulocyte activation, and neutrophil-mediated immunity. While proteins were enriched in muscle filament sliding, muscle cell development and differentiation showed decreased levels of phosphorylation (Figure 2A,B and Supplementary Table S1). Of note, cytoskeleton organization was enriched in both upregulated sites and downregulated sites while inflammation-related biological processes were only enriched in upregulated sites. Most proteins identified are tendon/muscle assemblies, be those with increased phosphorated p-sites or with decreased p-sites (Figures 2C,D). With respect to molecular function, a significant enrichment in integrin binding was witnessed for upregulated p-site proteins. In addition, the rest are enriched in basic muscle/tendon functions (Figures 2E,F).

### Pathway Analyses

**FIGURE 3:**
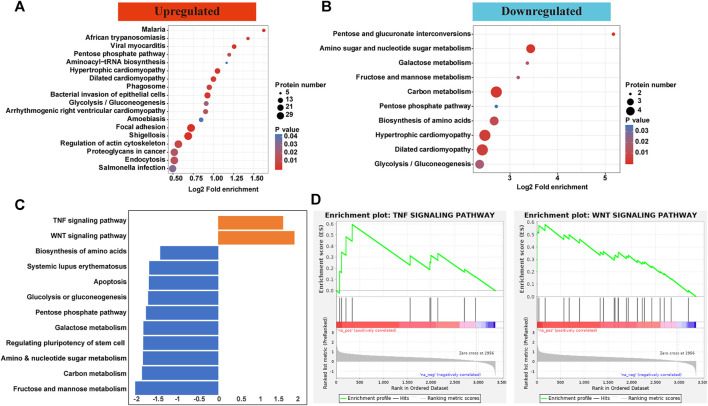
**(A,B)** KEGG pathways showed that proteins with upregulated p-sites were enriched microbe invasion reactions including malaria, African trypanosomiasis, viral myocarditis, amoebiasis, shigellosis, and salmonella. As for those with downregulated p-sites, proteins were enriched in metabolism including galactose, carbon, pentose, and glycolysis. **(C,D)** GSEA showed that most pathways enriched for proteins with decreased level of p-sites were similar with those enriched in KEGG pathway. Of note, two pathways for proteins with increased level of p-sites were exclusively identified by GSEA, which are TNF signaling pathway and Wnt pathway.

KEGG pathways showed that proteins with upregulated p-sites were enriched microbe invasion reactions including malaria, African trypanosomiasis, viral myocarditis, amoebiasis, shigellosis, and salmonella. As for those with downregulated p-sites, proteins were enriched in hypertrophic/dilated cardiomyopathy and metabolism including galactose, carbon, pentose, and glycolysis (Figure 3A, B). To further validate the responsible pathways, the GSEA approach, which took the quantification information into account, was applied. Most pathways enriched for proteins with decreased level of p-sites were similar with those enriched in KEGG pathway, focusing on metabolism. Considering that downregulated proteins and p-sites only contributed a minor part in this phosphorylation atlas (Figure 1A), we paid more attention to the upregulated ones. Of note, by GSEA, two pathways for proteins with increased level of p-sites were exclusively identified by GSEA, which are TNF signaling pathway and Wnt pathway (Figures 3C,D and Supplementary Table S2, S3, S4). Specifically, proteins including intercellular adhesion molecule 1 (ICAM1), Nuclear factor kappa-B (NF-κB), E3 ubiquitin-protein ligase Itchy homolog (ITCH), and Dynamin-1-like protein (DNM1L) were identified to be enriched in TNF signaling pathway. Protein kinase C alpha type (PKCα), Chromodomain-helicase-DNA-binding protein 8 (CHD8), and Calcium/calmodulin-dependent protein kinase type II subunit delta (CaMKII-δ) were found in our dataset to be enriched in Wnt pathway.

### Inferring Protein Kinase Identification

**FIGURE 4:**
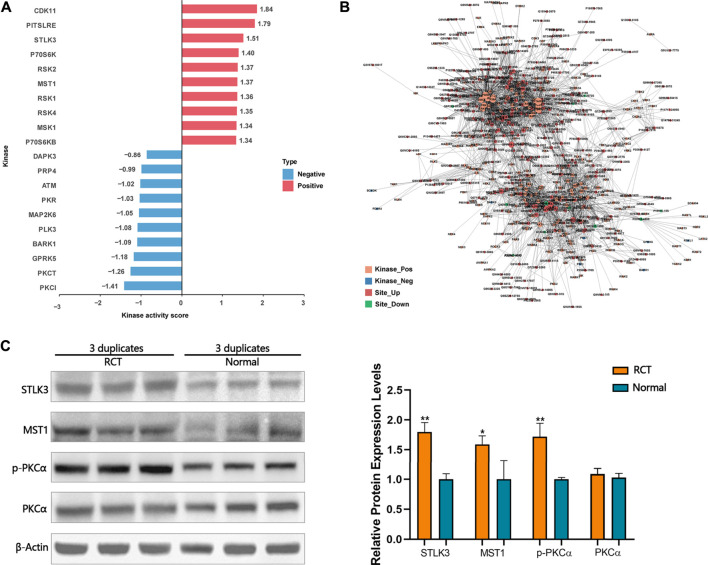
**(A)** Some activated and inactivated protein kinase were predicted by iKAP. **(B)** According to the kinase-site phosphorylation network, phosphorylation levels of PKCα that was enriched in Wnt pathway at T497 were positively correlated with STLK3 and MST1. **(C)** Western blotting validation. Differential expression levels of STLK3, MST1, and *p*-PKCα in RCT and normal tissues were verified using western blotting analysis. Paired samples *t*-test was used. *p < 0.05, **p < 0.01.

A novel computational method, iKAP (Chen et al., 2017), for inferring protein kinase activities from a weighted kinase-site phosphorylation network was applied. CDK11, PITSLRE, STLK3, P70S6K, RSK2, MST1, RSK1, RSK4, and P70S6KB were predicted to be activated within the tendinopathy site. DAPK3, PRP4, ATM, PKR, MAP2K6, PLK3, BARK1, GPRK5, PKCθ, and PKCι were predicted to be inactivated within the tendinopathy site (Figure 4A). Of note, according to the kinase-site phosphorylation network, phosphorylation level of PKCα that was enriched in Wnt pathway at T497 was positively correlated with STLK3 and MST1 (Figure 4B). Then, western blotting was performed for further verification (Figure 4C). However, the accurate regulatory relationship between these proteins in the pathology of RCT are further required.

## Discussion

Protein phosphorylation is of great importance for the coordination of cellular and organic functions including the regulation of metabolism, proliferation, apoptosis, subcellular trafficking, inflammation, and other important physiological processes. Here in this study, we provide a global phosphorylation atlas of proteins within the pathological site of rotator cuff tendinopathy by using human samples. Significant changes of phosphorylation levels for a considerable number of proteins have been witnessed. GO enrichment analyses have shown that proteins with upregulated p-sites mainly functioned in neutrophil-mediated immunity while those with downregulated p-sites functioned in muscle development. Furthermore, pathway analysis identified that NF-κB–related TNF signaling pathway and PKCα-related Wnt signaling pathway were associated with RCT pathology. At last, a weighted kinase-site phosphorylation network was built to identify potentially core kinase, which has found STLK3 and MST1 to be positively correlated with the activation of Wnt pathway found by pathway analysis.

Except for the specific findings about the pathways or responsible proteins, there was a fascinating discovery in the phosphorylation motif analysis (Figure 1D and Figure 2E). Analysis of the serine/threonine p-sites revealed that the phosphorylation motif showed a strong preference for proline (P) in the adjacent 6 positions either upstream or downstream. Phosphorylation motifs are consensus amino acid patterns around the phosphorylation sites. The discovery of these types of motifs can provide more precise rules to guide the recognition of kinase–substrate interactions. One of the most well-known motifs is proline-directed protein kinases, which are a subclass of protein serine–threonine kinases that phosphorylate proteins on a serine/threonine residue that is immediately preceding a proline residue. Proline-directed protein kinases include cyclin-dependent protein kinases, mitogen-activated protein kinases (MAPKs), glycogen synthase kinases, and Jun N-terminal kinases (Lu et al., 2002), playing an essential role in the regulation of cellular processes such as cell proliferation and differentiation. Here, we find this special p-site with proline residues both upstream and downstream. Does this pattern refer to another specific kind of kinases? This is of great interest for further study.

Much controversy has remained regarding the inflammatory mechanisms in tendinopathy, given that no classic inflammatory event including redness, tenderness, skin changes, or raised systemic inflammatory markers in blood has been witnessed (Rees et al., 2014). In addition, advances in tendon biology have increasingly recognized the lack of observation of an acute inflammatory infiltrate. Nonetheless, as research in tendinopathy pathophysiology has firmly identified a chronic degeneration of the matrix microenvironment, it is now increasingly evident that inflammatory responses are continuously activated within the tendon matrix microenvironment during tissue injury and contribute to dysregulated homeostasis (Millar et al., 2017). However, advancements in cellular profiling using genetic and molecular tools enabled research demonstrating the presence of mast cells, granulocytes, macrophages, T cells, and B cells in both acute and chronic human tendinopathic tissues (Arvind and Huang 2021). In this study, we identified p-sites of proteins involved in neutrophil reaction that were upregulated.

TNF plays a vital role in stimulating a variety of signaling pathways, including both MAPKs and nuclear factor kappa B (NF-κB pathways) (Akiyama et al., 2004). Subsequently, MAPK and NF-κB are both vital players in processes including inflammation, MMP secretion, apoptosis, and ossification in tendinopathy. Prior research by Han et al. (2017) demonstrated that TNF signaling contributed to the suppression of proliferation of tendon-derived stem cells. In addition, TNF-α was found to express within the inflamed and scarred site of tendon and mediate tendon degeneration (Hosaka et al., 2005; Schulze-Tanzil et al., 2011). Thus, there has been one experimental medicine trial using a fully humanized anti-TNF monoclonal antibody, adalimumab, which was originally designed to reduce pain and swelling due to certain types of arthritis, to treat a group of 10 athletes with symptomatic unilateral tendinopathy for more than 6 months (Fredberg and Ostgaard 2009). Walking pain showed a trend toward improvement at 7 days and 12 weeks post-treatment. Pain at rest was significantly reduced after 7 days and again showed a trend (*p* = 0.07) toward continued improvement at 12 weeks. However, given that multiple cellular processes are governed by MAPK pathway, side effects including rash, infection, and hypertension are common when using this agent (Scott and Kingsley 2006). Here in this study, only NF-κB–related TNF signaling pathway was enriched with pathological region in RCT patients. A further agent design through specifically targeting NF-κB may help reduce the chance of the aforementioned side effects (Abraham et al., 2019). Interestingly, a recent study focused on the expression levels of TNF pathway molecules in RCT patients with different clinical features (sex, age at admission, body mass index, the presence of night pain, previous treatment) (Struzik et al., 2021). The levels of expression varied from patient to patient and no exact relationship was found, which reminded us that there might be also other mechanisms except TNF pathway.

There were some limitations in this study for not considering the different disease causes such as chronic injuries and drugs. In our candidates, there were two statin-treated patients. Eliasson et al. (2017, 2019) found that statin could reduce the mechanical properties of tendon constructs and increase the risk of tendinopathy. However, no subgroup analysis was performed between statin-treated and non-treated patients on account of the small amount of each human sample. Even if the damaged tendon and the undamaged control sample were harvested from the same patient, which can help minimize the variance to some degree, the bias did exist. Still and all, in this paper, we tried to search for a common potential mechanism in the perspective of phosphorylation no matter what the disease-inducing factor was.

Research by Kishimoto et al. (2017) reported that activation of Wnt/β-catenin signaling suppressed expressions of tenogenic genes of Scleraxis (Scx), Mohawk (Mkx), and Tenomodulin (Tnmd) in rat tendon–derived cells isolated from the Achilles tendons of 6-week-old rats. All three genes are vital for tendon development, maintenance, and regeneration (Yang et al., 2019; Wong et al., 2020; Best et al., 2021). This suggests that an inhibition of Wnt/β-catenin signaling may represent a novel therapeutic target. However, no human sample–derived data have been present. Here, we showed that an increase in PKC-related Wnt pathway activation was correlated with RCTs, which may represent a prior neglect mechanism of tendinopathy. In addition, STLK3 and MST1, two protein kinases, may be associated with this activation. Therefore, potential therapeutic agent design may take into account on targeting this pathway. However, further experiments are required. What is more, except for RCT itself, associated complications such as muscle atrophy also bother people and lack clear mechanism (Liu et al., 2012). More attention and effort are needed in this research area.

## Data Availability

The datasets presented in this study can be found in online repositories. The names of the repository/repositories and accession number(s) can be found below: ProteomeXchange via the PRIDE database. The project accession is PXD029866.
